# Study on clinical nursing pathway to promote the effective implementation of sepsis bundle in septic shock

**DOI:** 10.1186/s40001-021-00540-8

**Published:** 2021-07-06

**Authors:** Chun-Xia Liu, Xian-Lei Wang, Kun Zhang, Gui-Zhen Hao, Wei-Yan Han, Yi-Qing Tian, Lan Ge, Li-Min Shen

**Affiliations:** Department of Critical Medicine, Hebei General Hospital, No, 348 Heping West Road, Shijiazhuang, 050051 Hebei China

**Keywords:** Clinical nursing pathway, Septic shock, Cluster treatment, Effective implementation, Intensive care

## Abstract

**Background:**

There is still a certain gap between the effective implementation and requirements of sepsis bundle. Our aim is to establish the clinical nursing pathway of the cluster treatment of septic shock in the Intensive Care Unit and promote effective implementation of the cluster treatment of septic shock.

**Methods:**

By means of evidence-based method, quality control index requirements and on-site investigation, the implementation process of clinical nursing pathway of the cluster treatment within 6 h of diagnosis of septic shock was established.

**Results:**

After the implementation of clinical nursing pathway, the completion rate of septic shock cluster treatment was 81.4% (66.4%) in 1 h, 89.4% (77.0%) in 3 h, 95.5% (82.3%) in 6 h (*P* < 0.05), which was significantly improved in the experimental group compared with the control group.

**Conclusions:**

The clinical nursing pathway of septic shock cluster treatment is guided by evidence-based nursing, which emphasizes standardization and standardization of septic shock cluster treatment nursing under the guidance of the guideline, and can promote the effective implementation of septic shock cluster treatment, significantly improve efficiency of septic shock treatment and the quality of medical care.

## Background

Septic shock is also known as infectious shock. The study of ICON provided global epidemiological data on 10,069 Intensive Care Unit (ICU) patients, confirming that 2973 (29.5%) patients had sepsis during admission or ICU stay. Among sepsis patients, the ICU mortality rate was 25.8%, and the hospital mortality rate was 35.3%, which was much higher than that of the general ICU population (ICU mortality rate was 16.2%; hospital mortality rate was 24.2%) [1]. Surviving Sepsis Campaign: International Guidelines for Management of Severe Sepsis and Septic Shock was first published in 2004 [2], and updated in 2008 [3], 2012 [4] and 2016 [5]. The overall goal of SSC is to reduce mortality of severe sepsis and septic shock. Active participation in SSC and improved adherence to guidelines are associated with the reduction in sepsis-related mortality [6]. Adherence to SSC guidelines can be promoted through the use of SSC sepsis bundle, including those completed at a specific time after the diagnosis of sepsis. However, the overall adherence in the process of sepsis bundle is low, and there is a large difference in the reaching rate among sepsis bundle items recommended by the guidelines [7]. There is still a certain gap between the effective implementation and requirements of sepsis bundle.

Clinical pathway is to formulate a standardized workflow according to the expected length of hospital stay of a certain disease, and conduct procedural and standardized management of medical and nursing behaviors, so as to improve the quality of medical care, shorten the length of hospital stay, and reduce medical costs [8]. It turns the terminal management of hospital quality control into link management. We have listed all the medical care contents involved and required by patients within 6 h after they were diagnosed as septic shock in ICU. By using the form tick, we designed the clinical nursing pathway of sepsis, so as to better guide and supervise the clinical medical staff’s effective implementation of sepsis bundle in septic shock.

## Methods

### General data

Two hundred twenty-six patients with septic shock admitted to our hospital from March 2017 to March 2020 were divided into control group and treatment group with the random number table, with 113 cases in each group. The control group was routinely treated with sepsis bundle, while the treatment group was treated by clinical nursing pathway of sepsis bundle. There was no statistically significant difference (*P* > 0.05) between the two groups in terms of age, gender, APACH II score, primary disease, etc., and they were comparable. See Table 1. This study was approved by the medical ethics committee of Hebei General Hospital (Scientific Research No. 108), and the Informed Consent Form was signed with the patient’s family members. The subjects can voluntarily terminate the study at any time without hindering further treatment.

**Table 1:** Comparison of general data between the two groups

Group	Cases	Sex (case)		Age (year)	APACH score	Protopathy (case)				
	(*n*)	Male	Female			Abdominal infection	Pulmonary infection	Bloodstream infection	Urinary tract infection	Other infections
Control group	113	62	51	77.58 ± 8.74	21.65 ± 4.85	54	22	15	11	11
Experience group	113	60	53	78.34 ± 7.93	22.78 ± 6.71	55	20	16	12	10
*χ*^2^/t		0.071		0.685	1.451	0.228				
P		0.790		0.494	0.148	0.994				

### Construction and implementation of clinical nursing pathway for sepsis bundle in septic shock

#### Construction of clinical nursing pathway for sepsis bundle in septic shock

The construction of pathway was under the guidance of evidence-based medicine, international guidelines, The Medical Letter 252 (2015) and the notification on printing and distributing six professional quality control indicators of anesthesia, etc. (2015 version), by National Health Commission of PRC. The clinical nursing pathway of sepsis bundle in septic shock is shown in Table 2. Attachment:

1. Objectives of 1-h sepsis bundle for septic shock: measure lactic acid, if initial lactate is more than 2 mmol/L, retest; collect blood culture samples before application of antibiotics; apply broad-spectrum antibiotics; apply 30 ml/kg crystalloid solution for target resuscitation under hypotension or lactate ≥ 4 mmol/L; apply vasopressor therapy to ensure that the mean arterial pressure (map) ≥ 65mmHg [9, 10].
2. The completion rate of 3-h sepsis bundle for septic shock: the completion rate of 3-h sepsis bundle for septic shock refers to the completion within 3 h after the diagnosis of septic shock: ➀ measure the concentration of lactic acid; ➁ blood culture before antimicrobial therapy; ➂ apply broad-spectrum antimicrobial drugs; ➃ apply 30 ml/kg crystalloid solution for target resuscitation under hypotension or lactate ≥ 4 mmol/L.
3. The completion rate of 6-h sepsis bundle for septic shock: based on 3-h sepsis bundle for septic shock, the 6-h sepsis bundle for septic shock should add: ➀ vasopressor should be immediately applied when hypotension has poor effect on target resuscitation; ➁ CVP and ScvO_2_ should be measured immediately under continuous hypotension after septic shock or lactate ≥ 4 mmol/L volume; ➂ lactate level should be measured repeatedly in patients with initial lactic acid higher than normal.

**Table 2:** Clinical nursing pathway form of sepsis shock cluster treatment

Time	1 h in ICU	3 h in ICU	6 h in ICU
Indicators to be completed	^a^Vital signs monitoring ^a^Evaluate the condition and implement emergency medical orders, such as endotracheal intubation ^a^Two fluid routes were established with indwelling needle ≤ 22G, and central venous access was established with doctors ^a^Invasive blood pressure monitoring by puncturing artery ^a^Collect all kinds of samples, especially blood gas, and measure the concentration of lactic acid ^a^Indwelling catheter to observe urine volume ^a^Blood culture before antibiotic treatment ^a^The patients were treated with broad-spectrum antibiotics ^a^Patients with hypotension or lactic acid ≥ 4 mmol/L were given 30 ml/kg crystal solution according to the doctor’s advice	^a^The project is not completed within 1 h, especially ^b^Measurement of lactic acid concentration ^b^Blood culture was performed before antibiotic treatment ^b^Give broad-spectrum antimicrobial therapy ^b^Low blood pressure or lactic acid ≥ 4 mmol/L was given 30 ml/kg crystalloid solution for target resuscitation ^a^The color, character and quantity of gastric juice were observed by indwelling gastric tube ^a^Blood glucose measurement ^a^Methods to prevent DVT, such as pneumatic therapy, subcutaneous injection of low molecular weight heparin, etc ^a^Treatment and nursing of primary disease ^a^Observation and nursing of sedation and analgesia ^a^Cluster nursing of ^a^CRBSI Cluster nursing of VAP ^a^Cluster nursing of CAUTI	^a^Project not completed within 3 h ^a^Give pressor ^a^Measure CVP ^a^Measure ScvO_2_ ^a^Repeated measurement of lactic acid level

#### Check the medical records

By reviewing the hospitalized medical records of patients with septic shock in our department, we can understand the current status, including the number of hospitalization days, costs, medical care content and implementation.

#### Expert consultation

Set up a clinical nursing pathway group in the department, invite the department director, doctors and nursing team leader to discuss together, and formulate the clinical nursing path framework for the sepsis bundle of septic shock.

#### Effective evaluation indicators of sepsis bundle in septic shock

Each time node completes all items as required, and the patient’s mean arterial pressure (MAP) ≥ 65 mmHg, central venous pressure (CVP) 8–12 mmHg, central venous oxygen saturation (ScvO_2_) ≥ 70% or mixed venous oxygen saturation (SvO_2_) ≥ 65%, urine volume ≥ 0.5 ml/kg/h after diagnosis of septic shock 6 h, the patient’s peripheral warm, skin mottling turns better.

### Implementation of clinical nursing pathway of sepsis bundle for septic shock

#### Do a good job of training and guidance

To improve the awareness of all medical staff on the importance of timely and effective implementation of the clinical nursing pathway of sepsis bundle for septic shock. Train the importance, necessity, and implementation methods of septic shock and sepsis bundle through daily morning and evening shifts, morning lectures and questions, level training and business rounds.

The team leader on duty is responsible for communicating with the doctor, supervising and assisting the responsible nurses to follow the clinical nursing pathway. The head nurse supervises, and the head nurse and team leader conduct two-way quality control.

### Statistical methods

SPSS 17.0 statistical software was used for data analysis. Count data were tested by *χ*^2^ between the patients in the two groups at a specific time, measurement data were expressed as mean ± standard deviation (x ± s), and *t* test was used for comparison between groups. *P* < 0.05 shows the difference is statistically significant.

## Results

After the implementation of the clinical nursing path of sepsis bundle for septic shock, the target completion rate of 1-h sepsis bundle of septic shock increased from 66.4 to 81.4%, and the completion rate of 3-h sepsis bundle of septic shock increased from 77 to 89.4%, the completion rate of 6 h sepsis bundle of septic shock increased from 82.3 to 95.5%, the treatment group was significantly higher than the control group, *p* < 0.05, the difference was statistically significant. See Table 3, 4 and 5 for details.

**Table 3:** Comparison of 1-h completion rate of bundle therapy for septic shock in cases (%)

Group	Total number (cases)	Number of completed (cases)	Number of incomplete (cases)	Completion rate (%)
Control group	113	75	38	66.4
Experience group	113	92	21	81.4
*χ*^2^ value				6.6288
*P* value				0.010

**Table 4:** Comparison of 3-h completion rate of bundle therapy for septic shock in cases (%)

Group	Total number (cases)	Number of completed (cases)	Number of incomplete (cases)	Completion rate (%)
Control group	113	87	26	77.0
Experience group	113	101	12	89.4
*χ*^2^ value				6.2004
*P* value				0.013

**Table 5:** Comparison of 6-h completion rate of bundle therapy for septic shock in cases (%)

Group	Total number (cases)	Number of completed (cases)	Number of incomplete (cases)	Completion rate (%)
Control group	113	93	20	82.3
Experience group	113	108	5	95.5
*χ*^2^ value				10.1194
*P* value				0.001

## Discussion

Septic shock is a medical emergency and should be treated and resuscitated immediately. The primary task of doctors and health care providers is to protect patients from harm, and sepsis bundle can help us to do this [11]. However, in the treatment process of patients with septic shock, the overall adherence of the sepsis bundle recommended by the guidelines was 77.25%, and there was a large difference in reaching rate among items. The reaching rate of 6-h early goal-directed therapy (EGDT) was only 66.67% [12]. The main reasons that affect the effective implementation of sepsis bundle for septic shock are that the medical staff, especially nurses, do not understand the guidelines, their time concept, awareness and adherence to sepsis bundle are poor, the nurse–bed ratio is low, and the time required for improving the medical records when patients transfer to another department leads to the delay of medical records transfer and the extension of medical orders and execution time [13]. The clinical nursing pathway helps nurses overcome the difficulties and implement the treatment and nursing measures at each time node according to the requirements. In each time period, the nurses should check the work completion of the previous stage according to the clinical nursing pathway table, and take effective measures to remedy the projects not completed on time. As a result, with the extension of time, the completion rate of sepsis bundle for septic shock gradually increases.

As an industrial management method, critical path was widely applied in American industry around 1950. In the medical field, DRG/PPS (Diagnosis Related Groups/Prospective Payment Systems) was introduced into the United States in 1983. Then, in 1985, Karen Zander from New England Medical Center in Boston introduced the method of critical path into the nursing of inpatients. In Japan, since about 1992, the clinical pathway has been centered on the Japanese medical management association and the Japanese clinical pathway society, as the enrichment of informed consent, the development of team medicine, the medical reform centered on patients, the saving of medical resources and the improvement of safety have been recognized and carried out to improve the medical quality. Clinical pathway is very necessary to promote efficient diagnosis and treatment. It can provide standard treatment and management systematization, improve the coordination of medical staff, save medical resources, and improve medical safety and patient satisfaction. Compared with the doctor-centered clinical pathway, the clinical pathway with the ward supervisor as the center has more continuity and can become a sound path activity. Clinical nursing pathway makes nurses’ work change from passive to active, cooperating with doctors, the whole process of diagnosis and treatment can reflect the opinions of nurses. Clinical nursing pathway can also deepen nurses’ understanding of the significance and results of examination or disposal, reduce medical errors, facilitate early detection of abnormalities, continue nursing care even for new nurses, promote team medical care, enhance communication with patients, and improve the trust of doctors and patients [14, 15].

After the implementation of the clinical nursing pathway of sepsis bundle for septic shock, the completion rate of 1-h, 3-h, 6-h sepsis bundle of septic shock was significantly improved, but it did not reach 100%, The reasons are as follows: patients’ economic difficulties or doctors’ working habits lead to difficulties in target monitored volume recovery; patients have received rehydration treatment before they are transferred to ICU; CVP measurement and ScvO_2_ monitoring can not be implemented due to femoral vein catheterization in emergency or other reasons or patients’ introduction of peripherally inserted central catheter [13]. To treat septic shock, multidisciplinary cooperation, early identification and diagnosis, predictive monitoring, fluid infusion and catheterization are needed to ensure the effective and correct implementation of sepsis bundle for septic shock [16].

## Conclusion

The clinical nursing pathway of sepsis bundle for septic shock is guided by evidence-based nursing. It emphasizes the standardization and standardization of nursing care under the guidance of the guideline, which can promote the effective implementation of sepsis bundle for septic shock and improve the satisfaction of doctors to nursing behavior and nursing work. It has important application value in promoting the unity of doctors and nurses, mobilizing the enthusiasm of nurses, reducing errors and improving the level of hospital service. It can significantly improve the treatment efficiency of septic shock and the quality of medical care.

## Data Availability

The datasets used and/or analyzed during the current study are available from the corresponding author on reasonable request.

## Abbreviations

ICU: Intensive care unit
MAP: Mean arterial pressure
CVP: Central venous pressure
ScvO_2_: Central venous oxygen saturation
SvO_2_: Mixed venous oxygen saturation
EGDT: Early goal-directed therapy
